# The complete mitochondrial genome of the blue runner, *Caranx crysos* (Mitchill, 1815) (Teleostei: Carangidae)

**DOI:** 10.1080/23802359.2021.1917319

**Published:** 2021-04-26

**Authors:** A-Young Jeon, Ji-Hyun Lee, Sapto Andriyono, J. Adonis Zuweh, Hyun-Woo Kim

**Affiliations:** a Department of Marine Biology, Pukyong National University, Busan, Republic of Korea; b Department of Marine, Fisheries and Marine Faculty, Universitas Airlangga C Campus Jl. Mulyorejo Surabaya East Java, Surabaya, Indonesia; c National Fisheries and Aquaculture Authority, Monrovia, Liberia

**Keywords:** Carangidae, *Caranx crysos*, mitogenome, next-generation sequencing

## Abstract

*Caranx crysos* was collected from offshore of Sierra Leone and its complete mitochondrial genome was determined using next-generation sequencing (NGS). The circular mitogenome encoded a typical 37 genes, including 13 protein-coding genes (PCGs), 2 ribosomal RNA genes (12S rRNA and 16S rRNA), and 22 tRNA genes. An unusual start codon (GTG) was identified for the *COX1* gene, and incomplete stop codons (T–/TA–) were found in seven genes, including *ND2*, *ND3*, *ND4*, *COX2*, *COX3*, *ATP6*, and *CytB*. All tRNAs were predicted to fold into the typical clover-leaf structures, except for tRNA^Ser-GCT^, which lacks the D-arm. *C. crysos* formed a monoclade with the tree other species belonging to the genus *Caranx*, apart from the others. Among them, *C. crysos* was most closely related to *Caranx melampygus* and *Caranx tille*. The mitogenome sequence of *C. crysos* provides information for a better understanding of evolutionary relationships, systemic, and mitogenomic study within the family Carangidae.

The blue runner, *Caranx crysos* is a marine fish in the family Carangidae, one of the most diverse taxa within the order Perciformes (Souza and Mafalda Júnior 2008). According to FishBase (www.fishbase.org), *C. crysos* is widely distributed in the Atlantic Ocean along the coastline ranging from eastern America to western Africa and Europe. Although *C. crysos* is considered one of the primary species in multiple fisheries, its taxonomic information is still not clearly established, making it difficult for scientific conservation and management (Duarte et al. 2017). We here first determined the complete mitochondrial genome of *C. crysos* collected from the African coastal waters as the species’ primary genetic information.

The specimen of *C. crysos* was collected from the offshore of Sierra Leone (8°00′00.0″N, 14°03′36.0″W) during a fish diversity survey. The identity of the specimen was confirmed based on morphological characteristics and by sequencing the *COX1* gene of the specimen. The voucher specimen and DNA are stored at the Marine Biodiversity Institute of Korea (https://www.mabik.re.kr/html/en/, Ha Yeun Song, and hysong@mabik.re.kr) under the number GR00004768. Mitochondrial DNA was extracted by Mitochondria DNA isolation kit (Abcam, Cambridge, UK), which was further sheared by Covaris M220 Focused-Ultrasonicator (Covaris Inc., San Diego, CA). TruSeq® RNA library preparation kit V2 was used to prepare a library for MiSeq sequencing platform (Illumina, San Diego, CA). Assembly of the raw reads and gene annotation was performed by Geneious^®^ version 11.0.2 software by mapping against a reference with medium-low sensitivity (Kearse et al. 2012). The loci and structures of the 22 tRNAs were predicted by tRNAScan-SE software (Lowe and Chan 2016). A phylogenetic tree was built with nucleotide sequences of 13 protein-coding genes (PCGs) from the eleven mitogenome sequences in the subfamily Caranginae using a maximum likelihood (ML) algorithm implemented in the MEGA version 7.0 program, in which the GTR + GAMMA substitution model and 1000 bootstrap replicates were employed (Kumar et al. 2016). *Seriola quinqueradiata* (GenBank accession AB517556) in the subfamily Naucratinae was designated as an outgroup.

The complete mitochondrial genome of *C. crysos* (MW435597) was 16,595 bp in length, which encoded 13 PCGs, 2 ribosomal RNA genes (12S rRNA and 16S rRNA), and 22 tRNA genes. The control region was located between tRNA^pro^ and tRNA^phe^, while the putative origin of light-strand replication (O_L_) was found within a cluster of five tRNA genes (WANCY). A slightly higher A + T ratio to G + C (1.17) was identified. Among 13 PCGs, 12 contained a typical start codon (ATG), except for the *COX1* gene (GTG). Incomplete stop codons (T–/TA-) were identified in seven genes, including *ND2*, *ND3*, *ND4*, *COX2*, *COX3*, *ATP6*, and *CytB*. With the exception of ND6, all the other PCGs were encoded on the light strand. Predicted tRNAs varied in size from 68 to 75 bp, and most of them form typical clover-leaf structures, while tRNA^Ser-GCT^ fold without the D-arm as shown in metazoan mitochondrial tRNAs (Watanabe et al. 2014).

The phylogenetic analysis supported the monophyly of the subfamily Caranginae (Figure 1). *C. crysos* was fully resolved in a monoclade with the three other species belonging to the genus *Caranx,* consistent with a previous analysis (Reed et al. 2002; Near et al. 2012). Within the genus, *C. crysos* was most closely related to *Caranx melampygus* and *Caranx tille*. The mitogenome sequence of *C. crysos* provides information for a better understanding of evolutionary relationships, systemic, and mitogenomic study within the family Carangidae.

**Figure 1.**
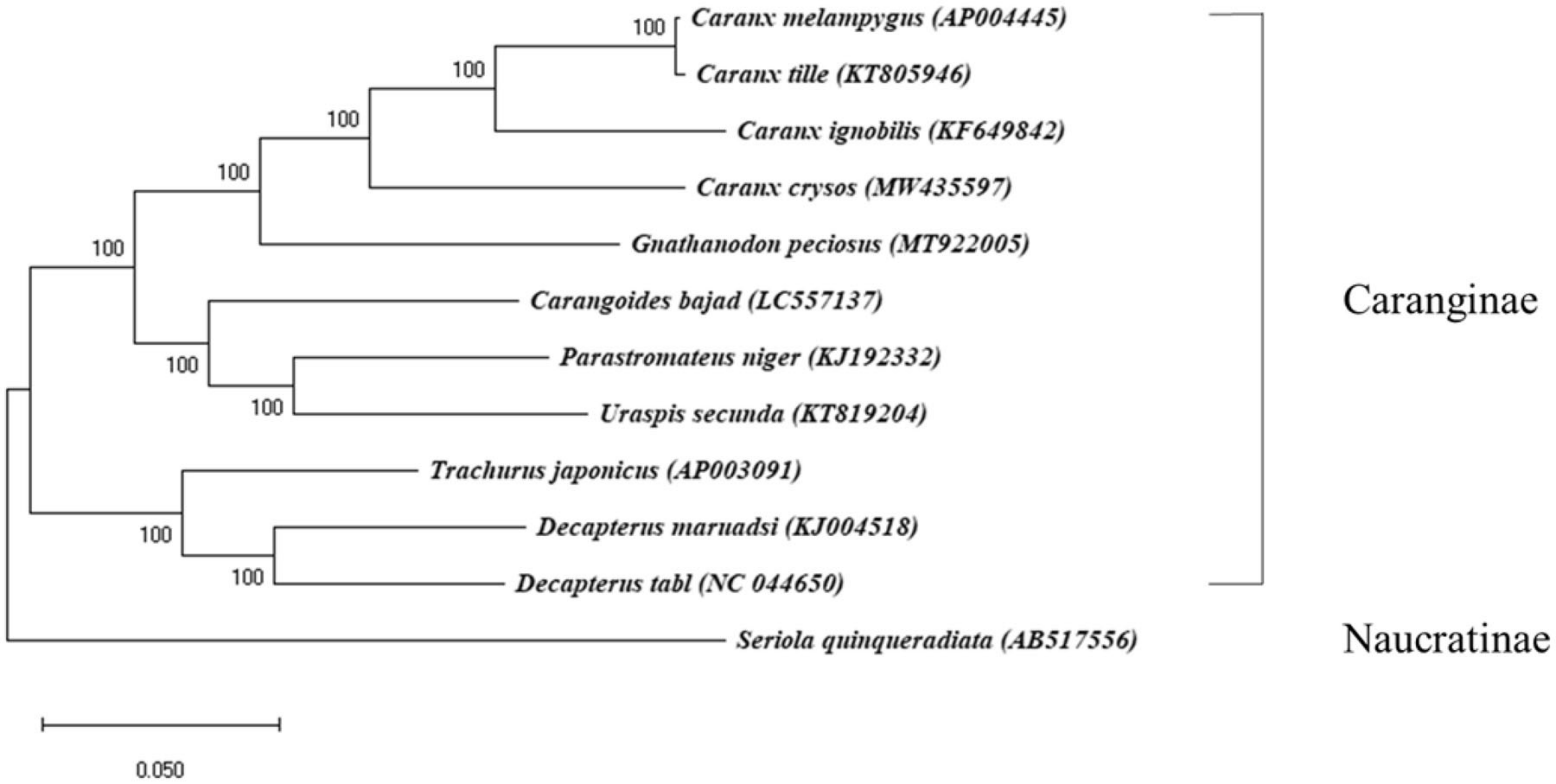
A maximum likelihood (ML) tree of 12 mitogenomes in the family Carangidae. In the ML tree, node confidence was estimated with 1000 bootstrap replicates. GenBank accession numbers are shown next to each species name. *Seriola quinqueradiata* was used as an outgroup and the present result of *Caranx crysos* is marked by an asterisk.

## Data Availability

The genome sequence data that support the findings of this study are openly available in GenBank of NCBI at [https://www.ncbi.nlm.nih.gov] (https://www.ncbi.nlm.nih.gov/) under the accession no. MW435597. The associated BioProject, SRA, and BioSample numbers are PRJNA706539, SRR13855214, and SAMN18137722, respectively.
